# Special Measures for Quality and Challenged Providers: Study Protocol for Evaluating the Impact of Improvement Interventions in NHS Trusts

**DOI:** 10.15171/ijhpm.2019.100

**Published:** 2019-11-16

**Authors:** Naomi Fulop, Estela Capelas Barbosa, Melissa Hill, Jean Ledger, Christopher Sherlaw-Johnson, Jonathan Spencer, Cecilia Vindrola-Padros, Steve Morris

**Affiliations:** ^1^Department of Applied Health Research, University College London, London, UK.; ^2^Research Team, The Nuffield Trust, London, UK.; ^3^Department of Public Health and Primary Care, University of Cambridge, Cambridge, UK.

**Keywords:** Organisational Failure, Special Measures for Quality, National Health Service (NHS), Mixed Methods Research

## Abstract

**Background:** Healthcare organisations in England rated as inadequate in terms of leadership and one other domain enter the Special Measures for Quality (SMQ) regime to receive increased support and oversight. There is also a ‘watch list’ of challenged National Health Service (NHS) providers at risk of going into SMQ that receive support. There is limited knowledge about whether the interventions used to deliver this support drive improvements in quality, their costs, and whether they strike the right balance between support and scrutiny. The study will seek to determine how provider organisations respond to these interventions, and whether and how these interventions impact organisations’ capacity to achieve and sustain quality improvements over time.

**Methods:** This is a multi-site, mixed methods study. We will carry out interviews at national level to understand the programme theory underpinning the interventions. We will conduct 8 NHS case studies to explore the impact and implementation of the interventions that form part of the SMQ and challenged providers programme. We will use a conceptual framework based on models of organisational readiness for change and draw on board maturity research for implementing quality improvement. We will also review the use of quantitative metrics and data for tracking the progress of improvements in quality of care and sustainability upon leaving SMQ, as well as the costs and benefits of the interventions through a cost-consequence analysis (CCA).

**Discussion:** High-quality interventions that successfully support struggling healthcare organisations are essential and an issue that is an international concern. Our study will allow a greater understanding of the programme theory, impact, and staff views and experiences of the SMQ and challenged providers regime. Formative feedback will be reported to key stakeholders.

## Background

There is an internationally recognised need for transparent, integrated, and timely processes for identifying quality and patient safety issues across healthcare systems.^1^ There may be indications of persistent performance issues in a healthcare organisation long before a crisis comes to the attention of the public and regulators. Attention has been placed on failing healthcare organisations, their characteristics and the factors (internal and external) that might lead to low performance. These include low leadership capability, lack of open culture, antagonistic external relationships,^2-4^ inadequate infrastructure, lack of a cohesive mission, and system shocks.^5^ A hierarchical culture and leadership focused on avoiding penalties and achieving financial targets - rather than a patient-centred mission - are characteristics identified in many failing organisations. High-quality interventions capable of helping struggling healthcare organisations to improve are essential.^5^

The Special Measures for Quality (SMQ) regime is a targeted and time-limited regime in the National Health Service (NHS) in England agreed between the Care Quality Commission (CQC) and NHS Improvement (NHSI). The regime emerged following the Keogh Review into avoidable mortality in 2013.^6^ Trusts are put into SMQ only where serious care quality failings are identified and the leadership appear unable to resolve the problems without intensive support and external input.^7-9^ The SMQ regime provides trusts with oversight and interventions from NHSI, the national regulator, to help them address specific quality failings identified in CQC inspections. NHSI perceive SMQ as a support regime to bring about improvement (correspondence, October 2018). There is also a ‘watch list’ of challenged NHS providers at risk of entering SMQ that receive support. Unlike SMQ, the challenged provider list is not available in the public domain. Interventions for SMQ/challenged providers typically vary between trusts and may include appointment of an Improvement Director; review of the trust’s leadership capability; access to financial resources for quality improvement; an improvement plan, including options for diagnostic work on assessing medical engagement; buddying with other trusts and commissioning external expertise. These may be delivered in conjunction with other interventions, and within a context of significant senior leadership changes.

Studies of the SMQ regime have highlighted unintended consequences for trusts, such as difficulties with recruitment and retention, lowering of staff and patient morale, increases in financial costs, and external pressures placed on already burdened management systems.^10^ A recent evaluation of the CQC inspection regime categorises 8 types of regulatory impact arising from the inspection regime (Table 1).^11^ The impact of CQC inspections was found to vary considerably according to type and size of provider, although ‘directive,’ ‘stakeholder’ and ‘organisational’ influences appear most applicable to providers that are asked by the regulator to take immediate action to improve quality and enter SMQ.

**Table 1:** Eight Regulatory Impact Mechanisms

**Impact Mechanism**	**Description**
Anticipatory	Providers seek to comply in advance of regulatory interactions (eg, inspection).
Directive	Providers take direct actions as requested by the regulator. Legal consequences possible in cases of non-compliance.
Organisational	Providers instigate internal processes not explicitly related to directives on account of interaction with the regulator, such as addressing leadership or culture.
Relational	Influence of (human, interpersonal) interactions between regulatory staff and regulated providers.
Informational	Regulatory information on performance enters the public domain and informs decision-making.
Stakeholder	Other stakeholders take action and interact with the regulated provider.
Lateral	Regulatory interaction results in new inter-organisational actions (across boundaries), such as peer learning.
Systemic	Regulatory information on providers is used to identify wider issues in systems of care, beyond a single provider.

^a^Adapted from Smithson et al.^11^

### The Special Measures Regime

We analysed data supplied by NHSI on trusts that had entered SMQ since the regime began in July 2013 up to September 30, 2018. A total of 35 trusts entered SMQ; 4 trusts returned to SMQ (giving 39 episodes), 25 had exited SMQ, and as of September 30, 2018, there were 14 trusts in SMQ. The “watch list” of challenged providers was initiated in July 2015. These trusts receive interventions to prevent them entering SMQ. On September 30, 2018, there were 17 trusts on this list. Since July 2015, 44 trusts have been placed on this list, with 17 trusts leaving the list because they entered SMQ, and 1 trust left the list and subsequently returned. Fifty-nine trusts entered SMQ or the challenged providers list between July 2013 and September 2018. As of January 2019, there were 234 trusts in England, meaning that roughly one quarter of trusts have experience with SMQ or the challenged providers regime (Table 2). NHSI also manages a Special Measures for Finance programme (SMF), introduced in July 2016. SMF will not be examined in this study.

**Table 2:** The Types of NHS Trust Entering SMQ or on the Challenged Providers List (July 2013-September 2018)

**Trust Type**	**Number of Trusts Ever in SMQ or on Challenged Providers List**	**Trusts in SMQ (at September 2018)**	**Trusts on Challenged Providers List (at September 2018)**
Acute services only	33	7	9
Acute and community	18	4	7
Acute and mental health	1	1	0
Ambulance	2	1	0
Community and mental health	1	0	1
Mental health	4	1	0
Total	59	14	17

Abbreviations: NHS, National Health Service; SMQ, Special Measures for Quality.

## Study Aims

The study will analyse the responses of providers to the implementation of (*a* ) interventions for trusts in SMQ and (*b* ) interventions for challenged providers to determine their impact on these organisations’ capacity to sustain and achieve quality improvements. We will focus on the main interventions that NHSI has identified as forming part of the SMQ/challenged provider regimes:

1) appointment and use of an Improvement Director;
2) buddying with other trusts;
3) the opportunity to bid for central funding to spend on quality improvement.

We will also include any other interventions participating trusts identify as being part of the SMQ/challenged provider regimes and consider these interventions within a wider context of any leadership changes. We will draw on evidence from the academic literature (eg, on organisational failure, turnaround and performance) to explore these issues and apply a range of appropriate quantitative, economic, and qualitative research methods.

## Research Questions

1. What are the programme theories (central and local) guiding the interventions delivered to trusts in SMQ/challenged provider regimes?
2. How and why do trusts respond to SMQ/challenged provider regimes and the interventions within these regimes?
3. Which features of trusts in SMQ/challenged provider regimes, and their wider context, contribute to their differing performance trajectories?
4. What are the relative costs of the interventions and how do these compare with their benefits?
5. How are data used by trusts in SMQ/challenged provider regimes, and how does data contribute to their understanding of improvements in quality and service delivery, especially in areas where performance concerns have been raised by the CQC?
6. Do trusts in SMQ/challenged provider regimes find it more difficult to recruit and retain staff?

## Methods

### Study Design

**Figure 1:**
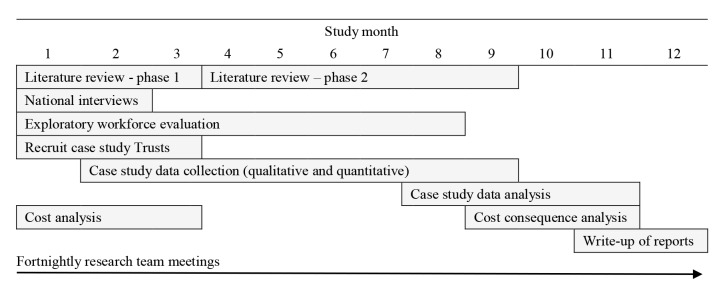
Study Timeline.

The study is being conducted by the National Institute for Health Research (NIHR) - Health Services and Delivery Research funded Rapid Service Evaluation Team (RSET); a 5 year research programme that aims to rapidly evaluate health and care service innovations to produce timely findings of national relevance and immediate use to decision-makers. This is a multi-site, mixed-methods study that will combine qualitative and quantitative approaches to analyse the implementation of interventions delivered to SMQ/challenged providers, and the impact of these interventions on trust performance, quality of care, patient experience, and costs. We will apply a conceptual framework based on previous models of organisational readiness for change and board maturity for quality improvement. To support the one year time frame data collection and analysis will follow a rapid research design^12^ involving: teams of field researchers, participatory approaches, and iterative data collection and analysis, with the research team meeting fortnightly to discuss progress and emergent findings (Figure 1).

Our study consists of 5 inter-related elements:

### 1. Literature Review Using Systematic Methods

We will conduct a rapid literature review of organisational failure in the public sector following rapid review methodology that uses a phased search approach.^13^ It will build on a recent review of organisational failure.^5^ Rapid reviews follow a systematic review approach, but some steps are adapted to reduce the time required to complete the review (ie, using large teams to review abstracts and full texts, and extract data; in lieu of dual screening and selection, a percentage of excluded articles are reviewed by a second reviewer, and software can be used for data extraction and synthesis^13^). The Preferred Reporting Items for Systematic Reviews and Meta-Analysis (PRISMA) statement will guide the reporting of the methods and findings.^14^ The review protocol is registered with PROSPERO (CRD42019131024).

#### Review Research Questions

##### Phase 1

1. How are ‘failing healthcare organisations’ defined?
2. What theoretical approaches have been used to explain organisational failure?
3. How is ‘organisational turnaround’ defined?
Which theoretical approaches have been used to study turnaround strategies (if any)?

##### Phase 2

1. What are the main interventions delivered to improve quality? Who delivers these interventions?
2. What are the programme theories guiding these interventions?
3. What are the main processes of implementation?
4. What is the impact of the implementation of these interventions? How are quality and performance monitored?
5. What is the sustainability of improvements produced by these interventions (if any)?
6. Have any of these interventions been evaluated? If so, how?
7. What are the costs and potential benefits of these interventions?

#### Search Strategy

##### Phase 1

Broad searches will cover literature from the fields of health services research, management and business studies to identify overarching themes on regulation, performance and quality improvement in healthcare organisations and the public sector (for an example of this approach see Ferlie et al^15^). The theoretical content from this literature will be used to develop a thematic framework of organisational failure and turnaround that will be used to inform the second phase.

##### Phase 2

Targeted searches will focus on interventions designed to address organisational failure and turnaround in healthcare and other public sectors such as education and local government settings and interventions delivered to high-performing organisations. We will search multiple databases: MEDLINE, CINAHL PLUS, EMBASE, and Web of Science for published literature. Results will be combined into Mendeley (Elsevier) and duplicates removed. Reference lists of included articles will be screened to identify additional relevant publications. We will hand search other relevant databases, such as the King’s Fund library, and search for relevant grey literature using Open Grey and TRIP.

#### Study Selection

Following rapid review methodology,^13^ one researcher will screen article titles, and 3 researchers will cross-check exclusions in the abstract and full-text phases. Disagreements will be resolved through discussion. Inclusion criteria are: (1) focus on delivering interventions in failing organisations, defined as not meeting required quality standards (self-defined or defined by external regulating organisation), (2) focus on delivering interventions in high-performing organisations (self-defined or defined by external regulating organisation), (3) describes empirical research, (4) describes research in healthcare and other public sector settings, (5) published in last 20 years, and (6) published in English.

#### Data Extraction And Management

Included articles will be analysed using a data extraction form developed in REDCap (Research Electronic Data Capture). The form will be piloted independently by 2 researchers using a random sample of 5 articles. Disagreements will be discussed until consensus is reached. The data extraction form will be finalised based on pilot findings.

#### Data Synthesis

Data will be exported from REDCap and the main article characteristics synthesised. Information entered in free text boxes will be exported from REDCap and analysed using framework analysis.^16^ The thematic framework developed Phase 1 will guide our exploration of themes.

#### Quality Assessment

We will use the Mixed Methods Appraisal Tool to assess article quality.^17^ Two researchers will rate these articles independently. Disagreements will be resolved through discussion. Inter-rater reliability will be calculated using the kappa statistic.^18^

### 2. Interviews at National Level

We will carry out 5-7 interviews with staff at national level. The research team will identify contacts within key stakeholder groups, including with NHSI, the CQC and the Department of Health and Social Care (DHSC) and ask for recommendations for individuals involved in the SMQ/challenged provider regimes. These individuals will then be invited independently to take part in a research interview. The purpose of these interviews is to better understand how the interventions deployed to support trusts are perceived by different stakeholders in relation to their programme theory/ies (ie, the underlying assumptions and expectations about the purpose of the intervention and the anticipated impact), and which interventions are viewed as being particularly effective, and under what conditions. The interview guide will cover 3 broad topic areas (1) aims of the SMQ/ challenged provider regimes; (2) policy and interventions; and (3) impact.

### 3. Multi-site, Mixed Method Case Studies

We will conduct 8 case studies (4 ‘high level,’ 4 ‘in-depth’). Case study research is common in management, business and organisational research and policy evaluations. Yin defines the case study as an ‘in-depth inquiry into a specific and complex phenomenon.’^19^ Case studies typically employ a range of data collection methods – quantitative, qualitative or a mixture of both – to ‘construct narratives of past events, or accounts of specific cases.’^20^

#### Conceptual Framework

Case studies will explore the implementation of interventions in SMQ/challenged provider trusts, reflecting on any observed changes in processes and outcomes reported across specified time points (eg, point of entry into, or exit from, SMQ). We will apply a board maturity framework developed in previous research, which found that boards with higher levels of maturity in relation to governing for quality improvement were able to effectively balance short-term (external) priorities against long-term (internal) investment in quality improvement and engage staff and patients in the process of change.^21^

In order to understand processes of quality improvement beyond board level, especially amongst “clear improvers” that exit SMQ and sustain change, we will use the concepts of absorptive capacity and dynamic capabilities from the strategic management literature to identify any routines or processes that have helped staff - from senior leaders to frontline clinicians - to learn from external information about performance and quality to sustain performance objectives.^22^ Absorptive capacity refers to the ability of organisations to acquire and exploit new information and knowledge and successfully transfer it internally – across organisational sub-units – to support learning and performance.^23^ Dynamic capabilities refers to patterned activities and routines that require dedicated resources and long-term commitment to effect impactful change.^24^ Applying such concepts will help to distinguish between evidence of incremental or ad hoc changes in trusts arising from externally driven SMQ interventions, and more radical or novel service innovations that improve quality and trust performance and have become embedded in new ways of working over time at trusts.

### Trust Sampling Framework

#### Inclusion criteria

- NHS trusts (ambulance, acute, mental health and/or community providers) placed in SMQ and/or the challenged providers regime *before* September 30, 2018.

#### Exclusion criteria

- Trusts placed in SMQ and/or the challenged providers regime (for the first time) *after* September 30, 2018.
- Trusts placed in SMF only and never in SMQ or the challenged providers regime.

Fifty-nine trusts meet our inclusion criteria (Table 2). Performance trajectories for these trusts were determined based on amount of time spent in SMQ/challenged provider regimes, and progress over time (Table 3).

**Table 3:** Descriptions of Performance Categories

**Performance Category** ^a^	**Performance Category Description**	**Trusts Matching Description** ^b^
‘Prolonged poor performers’	Trusts that have been in SMQ for 2 years or longer since the introduction of the regime, including those trusts that re-enter SMQ after a period of exit.	19
‘Poor performers’	Challenged providers who end up in SMQ.	12
‘Shorter term challenged providers’	Challenged providers who avoid entry into SMQ and have not previously been placed in SMQ. This may include trusts that merged with higher performing providers. They are or were ‘challenged’ for less than 2 years.	20
‘Clear performance improvers’	Trusts that have previously entered SMQ or been on the challenged providers list but later achieved a good or outstanding overall CQC rating, without re-entry into either regime.	9
Other trusts	Trusts that do not meet any of the other criteria (4 because they were ‘challenged’ for a longer time, and one because they left SMQ after a short period but have never been rated good or outstanding by CQC). These trusts were not sampled.	5

Abbreviations: CQC, Care Quality Commission; SMQ, Special Measures for Quality. ^a^Performance categories are neither exhaustive nor mutually exclusive. ^b^Some trusts fit multiple categories.

### Trust Sampling Strategy

**Figure 2:**
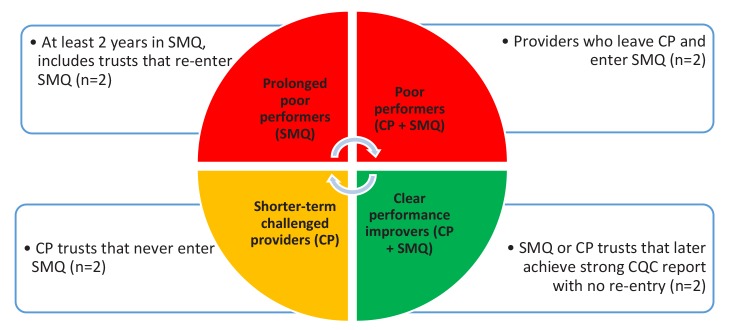
Purposive Sampling Model for Case Studies. Abbreviations: SMQ, Special Measures for Quality; CP, Challenged Providers; CQC, Care Quality Commission.

The overall objective of the case studies is to understand dynamics within trusts and their local contexts at different ends of the performance spectrum. To do this we will purposively sample 8 case study sites, with 2 sites from each performance category (Figure 2). We will attempt to account for provider size and type to ensure a range of organisations.

### (i) Qualitative Fieldwork

#### Data Collection

Qualitative fieldwork will combine semi-structured interviews, meeting observations, and documentary analysis (Table 4). Interviews and observations will document processes used to implement the interventions based on available data to plot a chronology of the quality improvement changes at each site. We will note internal (inner) and external (outer) contextual factors potentially influencing participation in the interventions, including senior level leadership changes and perceptions from the wider community and stakeholders in the local heath economy. We will remain open to understanding the interventions trusts perceive to be part of SMQ/challenged provider regimes, in addition to those identified by NHSI as being effective for driving change.

**Table 4:** Summary of Qualitative and Quantitative Data Collection at In-Depth vs. High-Level Sites

	**In-Depth Sites**	**High-Level Sites**
**Qualitative Components**		
Non-participant observation (eg, board meetings, operational meetings)	Yes Number of observations: 2 at each site (8 in total)	No
Interviews	Yes	Yes
	Participants: from across different organisational tiers + external stakeholders Sample size: 15 interviews at each site (60 in total)	Participants: from the top of the organisation + key external stakeholders Sample size: 8-10 interviews at each sites (32-40 in total)
Documentary analysis	Yes	Yes
**Quantitative Components**		
Trust use of quantitative information relating to quality of care	Yes	Yes
Tracking of outcome measures against improvement actions	Yes	No
Trust use of metrics to monitor impact of SMQ regime and challenged provider interventions	Yes	Yes

Abbreviation: SMQ, Special Measures for Quality.

To aid the quantitative analysis, we will study how people within trusts use data with an emphasis on whether and how data are used to track improvements in quality of care. To facilitate the economic analysis, we will query resource use and costs incurred by the different interventions, their perceived impacts on quality, and additional unintended consequences (positive or negative).

Documentary analysis will identify organisational strategies and variables that appear to indicate change over time (ie, since point of entry into SMQ) - such as shifts in organisational composition (eg, workforce numbers and vacancy levels) and changes in organisational structure (eg, new governance systems or mergers). This analysis will also seek to compare central and local theories guiding quality improvement efforts. Throughout the data collection process, the data will be summarised in the form of RAP (Rapid Assessment Procedures) sheets. The RAP sheets will facilitate consistency in data collection across 3 qualitative researchers and will allow the team to identify gaps in data collection that need to be addressed before the fieldwork ends.

### Sampling

#### Participants

We propose to use “vertical slicing” in the 4 ‘in-depth’ sites, conducting interviews across different organisational tiers as well as with external stakeholders, including patient groups. Up to 15 interviews will be completed at each in-depth case study site. The types of interviewees will depend on the context of each trust, but are likely to include divisional/clinical directors, and may include staff from a clinical unit which the CQC has flagged as ‘inadequate.’ In the 4 ‘high-level’ sites, we will conduct 8-10 interviews at the ‘top’ of the organisation and with key external stakeholders.

#### Non-participant Observations

We will observe public Trust board meetings and quality or performance-focused meetings at divisional level, after securing prior permission, at the 4 ‘in-depth’ sites and gaining individual consent from participants at the time of the meeting. We will use the board quality improvement maturity framework^21^ in our observations of boards and other relevant meetings to support analysis of observational data. We will focus on critical quality incidents or service issues where progress in quality improvement appears ‘transparently observable’ or where improvements are proving especially challenging for the organisations.^25^ Thus, we might study a particular clinical unit that has been flagged as in need for improvement in earlier CQC inspections or a new intervention the trust has introduced to support staff engagement in quality improvement, such as ‘quality huddles.’

#### Documents

We will collect documents on SMQ/challenge provider regimes produced centrally (eg, by the DHSC or NHSI), as well as those developed by trusts to operationalise improvement efforts and recommendations from the regulator. For example, we will examine relevant meeting minutes (eg, board meetings and operational units), strategic performance documents and business plans to help triangulate findings from interviews and observations.

### (ii) The Use of Data by Trusts

To complement the qualitative analysis, we will look deeper into the way data are being used by case study trusts, focussing specifically on how they monitor the impact of interventions and track quality improvements and whether they perceive that they have the capabilities and resources to do so effectively. We will also assess any changes in the way trusts use data once being placed into special measures or identified as “challenged,” including investing resources to support more accurate data collection and monitoring. Moreover, we will analyse whether, and how, trusts track progress against required improvements and these examples could offer helpful insights into whether trusts will be resilient to future challenges.

This work will link with the qualitative analysis described above in that trust interviews will provide on-the-ground insight into how being in special measures, or on the challenged provider list, influences their approach to the collection of data and how they monitor quality. They may also provide details on specific measures they are using and the qualitative interview topic guides will include questions focused on the trust’s use of information. Additional interviews will be conducted if necessary to gather specific information on data collection and monitoring of quality.

Other sources of information will be CQC inspection reports, documents produced by, or on behalf of, trusts (eg, Board reports, Quality Accounts), NHSI monthly monitoring of trusts and changes in their performance (eg, NHSI Single Oversight Framework segmentation) and, for wider context, the findings of the rapid literature review. These, in turn, may produce further lines of enquiry that could be followed up with the case study sites.

The analysis will include monitoring relevant improvement actions highlighted by these documents where they can be appropriately linked to outcomes observed in data. For example, if CQC raised concerns about a stroke service, then we would be interested in how the trust used data to track outcomes and to provide assurance that the quality of the stroke service is improving. There may also be evidence to suggest that pressures on some outcomes are related to performance of other providers in the local health system which we will investigate, where feasible. For some measures, data would be available to put the trust’s changes in outcomes within a national context, and possible sources include material published by the trusts (eg, Board papers), published statistics and patient-level records from Hospital Episode Statistics and national monitoring reports produced by NHSI (eg, monthly Single Oversight Framework segmentation spreadsheets). (Note: we have approval from NHS Digital covering all projects conducted by the REST.)

### (iii) Case Study Data Analysis

Triangulation of interview, observational, documentary and quantitative data will produce 8 local case studies that will be analysed thematically and comparatively, consistent with suggestions in academic literature on analysing processes of change in organisations^25-27^ and on receptive contexts for sustaining quality improvement in healthcare.^28,29^

### 4. Exploratory Workforce Evaluation

This additional quantitative component will explore relationships between workforce data and being in SMQ or on the challenged providers list. We will explore whether trusts find it more difficult to recruit and retain staff, or whether staff turnover increases after entering SMQ/challenged provider regimes. This will require trust-level workforce recruitment, retention, and turnover data from NHS Digital and information on vacancies and agency staff from NHSI. These data will be combined with trust inspection information from CQC, and details of time spent in any quality regime. Additional trust information such as size, financial situation, teaching status, measures of underlying patient need, or rurality will be considered. This proposed analysis is exploratory and subject to construction of a consistent and comparable workforce dataset and sufficient sample sizes to establish any statistical links. In any event we will be able to raise hypotheses that could be tested more robustly in future studies and reflected back to the case study sites for their qualitative insights.

### 5. Economic Analysis

The economic analysis aims to quantify the costs and benefits of different combinations of interventions used in SMQ/challenged provider regimes from an NHS perspective, using a cost-consequence analysis (CCA) approach. A CCA compares interventions in which the components of incremental costs (direct or indirect) and consequences (eg, knowledge, behaviours, processes) are computed and listed, without aggregating these results into a cost-effectiveness ratio.^30^ This approach enables one to look into process measures and qualitative findings in a quantitative manner, allowing for some insight as to how potential benefits compare to the cost of interventions.

A feasibility study for the economic analysis found that:

1. A CCA was feasible, but it would only be possible to evaluate different combinations of interventions, ie, it would not be possible to evaluate the benefits of each intervention individually. It would need to account for likely variation in the type and intensity of these interventions, eg, percentage of full-time equivalent (FTE) time the Improvement Director spends at the trust, different buddying models, varying receipt of funds spent in different ways. We will explore the impact of this variation on both costs and consequences.
2. Costs could be measured using resource use and unit cost data collected during the multi-site mixed methods study.
3. Consequences could be measured using qualitative data collected during the multi-site mixed methods study and/or combining it with quantitative data.

#### Cost Analysis

The first component of the economic analysis consists of a cost analysis, which is feasible as a standalone analysis, as it does not depend on qualitative findings from the case studies or quantitative analysis.

Each intervention will be costed using data collected during the case studies. We will calculate the costs of appointing an external Improvement Director based on salary costs plus other costs incurred, accounting for whether and how these costs may be shared across different trusts. We will identify the activities that typically occur with different models of buddying (eg, meetings, site visits, etc), and calculate the costs of these different models, including costs incurred by both the trust in SMQ and the ‘buddy’ trust. We will obtain information on the funds received to support improvement (challenged providers may access up to £200 000, while SMQ trusts may access up to £500 000), and identify how these monies, when received, are spent. For each intervention, we will also identify where the cost is incurred. We will also include opportunity costs as perceived by SMQ/challenged providers from an NHS perspective.

### Cost-Consequence Analysis

Based on the qualitative findings from the case studies, we aim to conduct a CCA that will evaluate the consequences of different combinations of interventions, considering type/intensity, and compare these with their costs. Consequences will be measured using qualitative data collected from the case studies, based on recurrent topics raised in the case studies’ emerging findings. These will be transformed into a meaningful scale, to quantify relative impacts of the intervention combinations. This approach allows for the findings of the process evaluation to be understood as a relative impact, which can be balanced against costs.

As there is likely to be between-trust variation in the type and intensity of the 3 main interventions, we will explore how consequences vary by type and intensity. Given that the measurement of both costs and consequences will depend on data collection from the case studies, the economic analysis will take place in 2 phases: (1) a cost analysis to take place in the initial months following access to selected NHS trusts and (2) a CCA following data collection in the case studies.

### Patient and Public Involvement

Two patient representatives provided feedback on the protocol and will provide ongoing review and feedback throughout the study (including dissemination). We presented the study topic to a local Research Advisory Panel, comprising patient representatives and members of the public. The Research Advisory Panel will provide programme level involvement, as distinct from individual evaluation-level and engagement activity. The Research Advisory Panel feedback informed protocol development, and additional feedback will be requested throughout the study. We will devise a local involvement and engagement strategy and when the site sample is defined, we will contact local patient groups, or individual key informers to obtain their views.

## Discussion

This mixed methods study will enable an analysis of the SMQ/challenged providers regimes and the interventions within them that have been set out by NHSI from the point of view of a wide range of stakeholders and multiple participating sites. The proposed research will inform our understanding of how trusts respond to the interventions and how they may impact on organisations’ capacity to achieve and sustain quality improvements over time. We will also gain insights into staff views and experiences with processes of change and improvement. Undertaking case studies of sites experienced with the SMQ/challenged provider regimes is a central component of the proposal. In addition to facilitating an analysis of the interventions delivered by NHSI, using a case study approach will also allow us to remain mindful that additional interventions may be deployed, and significant changes to leadership are frequently concurrent.

We anticipate generating lessons for trusts on responding to the interventions, such as what to prioritise and the organisational capabilities that have been found to support quality improvement. There will also be lessons for national bodies on how to support these trusts in light of the varied internal and external contexts of healthcare organisations facing performance difficulties. The information gathered in this study will also lead to recommendations on using routine and quantitative data to track quality improvements, including at system level, and set out the potential costs and benefits of individual interventions in the SMQ/challenged provider regimes. Research examining the interventions delivered by NHSI is limited and another key output from the study will be a conceptual framework that will to help evaluate the SMQ/challenged provider regimes in future. Our findings may also have wider applications to other regulated contexts, such as the education sector, and public sector management theory more broadly.

A key strength of the study is the mixed method approach that comprises a literature review, in-depth case studies, quantitative analyses that will be responsive to emerging themes from the case studies, and consideration of costs and potential consequences in an economic evaluation. There are, however, some potential limitations. The study duration of one year means that we will only have a partial view of the process and cannot study developments within the 8 case studies and their strategic responses to performance issues longitudinally. In addition, some data will be retrospective. It is also possible that access to case study sites may be constrained due to the sensitive nature of the research topic. The number of case-study sites may make it difficult to draw general conclusions about use of data, although our sampling is in line with many other high quality examples of case study research which apply mixed methods, and may have fewer than 8 organisations to compare. Within the trusts themselves, there may be problems accessing relevant data in which case we aim to be pragmatic and work with what we can obtain from national and publicly available datasets. Finally, the economic component of the study will not be able to disentangle individual effects from each intervention due to the complex nature and implementation unique to each participating site.

## Acknowledgements

We thank Christine Taylor (Department of Applied Health Research, UCL) for critical review of the manuscript and administrative support of the study. This report is independent research funded by the NIHR (Health Services and Delivery Research, 16/138/17 – Rapid Service Evaluation Research Team). NJF is an NIHR Senior Investigator. NJF, ECB, and SM are supported in part by the NIHR Collaboration for Leadership in Applied Health Research Care (CLAHRC) North Thames at Barts Health NHS Trust. The views expressed are those of the authors and not necessarily those of the NHS, the NIHR or the DHSC.

## Ethical issues

The UCL R&D Office and Ethics Committee reviewed the study protocol and materials. The study was classified as a service evaluation, not requiring research ethics committee approval. We will follow guidelines for data security, confidentiality and information governance. Data will be stored on a password protected UCL share drive that all research team members can access, sensitive or non-anonymised documents will be individually password protected. An informed consent process using participant information sheets and written consent will be used for recruitment to ensure informed and voluntary participation. We are aware of the sensitive nature of this research for organisations and individuals. The research team has experience in conducting research on similar sensitive topics. We will maintain the independence of the research and the anonymity of participants and organisations will be upheld.

## Competing interests

Authors declare that they have no competing interests.

## Authors’ contributions

NJF, CV-P, ECB, JL, CS-J, JS, and SM contributed to the conception and design of the research and drafted the manuscript. All authors contributed to revision of the manuscript and approve the final manuscript. NJF will oversee the full study; SM will oversee the economic analysis; CV-P will lead the rapid review; ECB will conduct the economic analysis; JL, CV-P, and MH will conduct the qualitative fieldwork and analysis; and JS and CS-J will conduct the quantitative analysis. All authors will contribute to integrating the findings.

## Authors’ affiliations

^1^Department of Applied Health Research, University College London, London, UK. ^2^Research Team, The Nuffield Trust, London, UK. ^3^Department of Public Health and Primary Care, University of Cambridge, Cambridge, UK.
